# Sleep, Health and Wellness at Work: A Scoping Review

**DOI:** 10.3390/ijerph14111347

**Published:** 2017-11-06

**Authors:** Nicola Magnavita, Sergio Garbarino

**Affiliations:** 1 Occupational Health Unit, Institute of Public Health, Università Cattolica del Sacro Cuore, Largo Gemelli 8, 00168 Rome, Italy; 2 Department of Neuroscience, Rehabilitation, Ophthalmology, Genetics and Maternal/Child Sciences (DINOGMI), University of Genoa, 16132 Genoa, Italy; sgarbarino.neuro@gmail.com; 3 Department of Health Sciences, Postgraduate School in Occupational Medicine, University of Genoa, 16132 Genoa, Italy

**Keywords:** sleep, wellbeing, workplace, occupational risk, environmental risk, sleep disorders, behavior, ageing, health promotion, lifestyle

## Abstract

Many occupational factors may interfere with sleep. Sleep disturbances can, in turn, endanger the health and safety of workers. This rapid review of the literature identifies the main factors that alter the quantity and quality of sleep, indicates the effects these alterations have on the wellbeing of workers and suggests some health promotion measures.

## 1. Introduction

Sleep has a vital effect on brain functions and many body systems. Studies on sleep regulation suggest that two distinct and separately regulated processes may exist: the homeostatic sleep-wake process S, and the circadian process C controlled by the circadian pacemaker [1,2]. Occupational factors can interact with both these mechanisms, inhibiting rest even when there is a need for sleep and altering biorhythms through the demand for anti-circadian activities. As a consequence, work-related sleep disorders are very common and may have significant short- and long-term effects on health and safety [3].

Not only environmental factors, but also lifestyles and diseases can be the cause of irregular sleep patterns. Lack of sleep or inadequate sleep are associated with a wide variety of unfavorable events: industrial and civil disasters, road accidents, distress and psychiatric conditions, drug abuse, increased mortality and morbidity, increased healthcare costs, direct economic costs, detrimental health effects and a reduction in overall wellness, performance and productivity [1,4].

While it is relatively easy to demonstrate the negative health effects in sleep-deprived workers, it is more difficult to quantify the reduction in their wellbeing. Consequently, the latter is often overlooked, but it should nevertheless be considered an equally important factor since wellbeing has a direct impact on physical and mental health. A recent European survey on health, age and retirement demonstrated that perceived wellbeing is inversely associated with the incidence of chronic obstructive bronchitis [5] and arthritis [6] in workers over a 9-year period, especially in men. There is also a significant relationship between wellbeing and general positive health indicators such as job satisfaction, work engagement and happiness and the quality of life, morbidity and productivity. Other studies have shown that vital aspects such as emotional stability, conscience, determination, control and optimism are positively correlated with wellbeing and inversely correlated with the incidence of depression, social isolation and loneliness, as well as with the prevalence of cardiovascular risk factors and chronic and degenerative diseases [7]. Enjoyment of life was also found to be negatively associated with mortality in the English longitudinal study on aging conducted on about 10,000 individuals between 2006 and 2013 [8].

In the complex relationship between wellbeing, health and productivity, sleep impairment can act as a moderator. Sleep disturbances may be both the cause and the consequence of reduced wellbeing and may therefore set up a vicious circle with relevant consequences for productivity and, in the longer term, the safety and health of workers.

In this paper, we intend to briefly review the main factors that can alter the amount and quality of sleep in workers and also discuss the effect sleep disorders have on workers’ wellbeing. We used the concept of “sleep” extensively, taking into account for their effects on wellbeing both sleep debt, which classically includes insufficient sleep, short sleep duration, unmet sleep needs, non-restorative sleep and other self-perceived sleep problems, and sleep disorders, including all the diseases reported in the 3rd International Classification of Sleep Disorders (ICSD-3) produced by the American Academy of Sleep Medicine [9]. We also looked at “worker” in a comprehensive manner, instead of splitting our analysis into different occupations and professional sectors. In our analysis, we adopted the point of view of the occupational physician who is tasked with supervising the health of workers at the workplace. In his or her work, the physician must consider not only the occupational risk factors, which result directly from the work, but also the environmental and individual ones that can interact with work, according to the holistic approach of occupational health [10]. For this reason, we preferred to report above all studies conducted in workplaces on active populations, and only when these were unavailable, we cited studies conducted on the general population, which however included workers.

In this review, we aimed to map rapidly the key concepts underpinning this research area and the main sources and types of evidence available, limiting the literature search to published literature on one database (PubMed), limiting inclusion criteria by the last 5 years, in English language. We had one person screening (NM) and another verifying excluded studies (SG). We did not conduct quality appraisal, and presented results as a narrative summary. We believe that this analysis is an essential requisite for implementing prevention and health promotion programs in the workplace.

## 2. Part I. Occupational Factors That Influence Sleep

Sleep physiopathology is influenced by a set of biopsychological factors that include age, gender, biotype, psychosocial state (presence of depression, stress, loneliness), socio-economic position, race and ethnicity. Moreover, since the duration of sleep is partially determined by behavior, cultural values, beliefs and practices may strongly influence sleep-wake variations [11]. A number of twin studies aimed at understanding factors contributing to variability in normal sleep-wake characteristics, demonstrated associations between sleep disturbances and emotional, behavioral and health-related problems as well as the existence of gene/environment correlation and interaction [12].

### 2.1. Shift Work, Hypnotype and Chronotype

Shift work is a vital component of our modern economy. Throughout the industrialized world, nearly a fifth of all employees are engaged in some form of non-traditional work pattern. Consequently, shift work is emerging as a social factor that causes illness in the working population. Beyond what is stated in the fundamental revision of literature conducted by Kecklund and Axelsson that is based on 38 meta-analyses and 24 systematic reviews demonstrating the association of shift work and insufficient sleep with chronic disease and accidents [13], we observe that shift work is also independently associated with numerous bad health indicators even in subjects who perform rigorous physical activity and who do not work at night. A recent study of more than 270,000 British workers demonstrated that shift work (not necessarily at night), is associated with several indicators of reduced wellbeing and traits of neuroticism. It is associated with obesity, depression and sleep disorders even in people who maintain a high level of physical activity [14].

Individual sleep patterns, e.g., the hypnotype (long and short sleepers) and the chronotype (circadian preference, morningness/eveningness) influence health problems associated with shifts. Occupational tasks interfere mainly with sleep duration, but sleep rhythm can also be altered. A substantial percentage of shift workers develop shift work disorder, a circadian rhythm sleep disorder characterized by excessive sleepiness, insomnia, or both as a result of shift work [15]. Loss of sleep and alterations in the circadian rhythm that disturb body functions play a central role in occupational illnesses caused by shift work [16].

Since sleep duration is an easily measurable parameter, it has been investigated much more than the quality of sleep. In a systematic review of studies on community-dwelling adults carried out between the 1960s and the 2000s [17], no consistent evidence was found to support the common assertion that adult sleep duration has declined over time. Sleep duration and health might have a U-shaped relationship: both short and long sleep durations are associated with a higher likelihood of healthcare use [18]. Inadequate sleep duration is a possible contributor to obesity and type 2 diabetes [19] and is associated with cardiovascular morbidity, even if this association is confounded by many psychological and socioeconomic variables [20]. Meta-analytic studies have shown that short-duration sleepers have an increased risk of all-cause mortality, as well as of cardiovascular-related and cancer-related mortality [21]. Long sleepers are also at increased risk of all-cause mortality [21]. However, the relationship between sleep duration and metabolic disorders is still controversial owing to methodological issues that hamper the epidemiological design of these studies [22].

Chronotype modulates the effects of the working schedule: early chronotypes have poorer and shorter sleep and show a greater degree of circadian misalignment during night shifts than late chronotypes. Conversely, late chronotypes have more sleep and more circadian alteration when working in morning shifts. Data from the United Kingdom Biobank project that enrolled more than 400,000 workers showed that long sleepers with evening preference had increased odds of tobacco use, sedentary behavior, and inappropriate diet than adequate sleepers with morning preference (reference group) [23]. The late chronotype is associated with poorer sleep quality in night-shift nurses and poor sleep quality is correlated in turn with frequency of musculoskeletal disorders [24]. Adopting a chronotype-based schedule can reduce circadian alteration and improve sleep and thus wellbeing, with potential long-term health and economic benefits [25].

### 2.2. Shift Work and Old Age

In recent years in all developed countries it has been necessary to significantly prolong the retirement age and limit early retirement opportunities [26], thus increasing the average age of the workforce and the share of older workers. Since, in most cases, this has occurred without changing work organization, one wonders what effect this has on the wellbeing of workers. In fact, both age and stress are associated with an increase in sleep problems. In the workplace these two factors, age and stress, can interact significantly, as reported in numerous studies. For example, in Australian miners, stress related to work, the lack of shift control and partner dissatisfaction due to shift work are related to sleep problems, but only in older workers. Conversely, lack of shift work control is not associated with sleep disorders in younger workers [27].

Shift work interferes with the quality of sleep. This alteration can become chronic and remain even after exposure has ceased. It has been reported that even after retirement older workers who worked in shifts have a worse sleep pattern than other retirees. In these former workers, polysomnographic studies have demonstrated the existence of a dose-response relationship between the duration of shift work and the frequency of altered sleep patterns [28]. A certain amount of time is needed to regain body regulation. The longitudinal observations in the GAZEL cohort indicate that sleep disturbances tend to decrease gradually but significantly after the cessation of work [29].

Studies conducted on long distance drivers indicate that older people experience greater fragmentation of sleep than their younger colleagues, or the general population. In adults, sleep fragmentation increases daytime sleepiness and worsens both performance and mood. Like shift workers, older drivers also report lower levels of sleep and this reduction is associated with worse performance [30].

Despite the common opinion that the elderly experience more problems in shift work than other workers, a systematic review of the literature revealed significant differences between the young and the elderly, especially as regards greater frequency of sleep disturbances in the latter, but failed to find strong evidence of a reduced tolerance of shifts in older workers [31]. This was probably due to the difficulty of isolating the effect of age from that of all the other competing risk factors in epidemiological studies.

### 2.3. Organization of Shifts and Duration of Rest

One of the factors that influence the risk associated with shift work is the way it is organized. Not all types of shift work are equally harmful. Irregular shifts, i.e., those beginning before 7 a.m. or 2 p.m., are associated with greater sleep problems, which in turn may lead to a higher prevalence of sexual function disorders and hormonal activity changes in workers [32].

The duration of the rest period, i.e., the interval between one shift and another is of great importance in reducing the amount and quality of sleep. A rapid return to work after an interval of less than 11 h between one shift and the next is associated with short sleep and fatigue during the following shift. A Swedish longitudinal study demonstrated that the short duration of the interval between the end of a work shift and the start of the next one is the principle factor in determining the quality and duration of sleep. A rapid return to work also decreases work satisfaction and increases fatigue and problems in family relationships. On the contrary, the frequency of night work was not associated with any of the aforementioned factors in a study by Dahlgren et al. [33].

In brief, it is not night work itself that causes problems, but being forced to perform work activities contrary to physiological rhythms over a prolonged period of time. Altering biorhythms impairs the function of the central nervous system and may also interfere with the circadian rhythm of cardiomyocytes, thus exerting a direct pathogenic effect on the heart [34].

A review of evidence concerning worksite interventions for shift workers concluded that some important preventive coping strategies for fatigue associated with shift work such as napping and exposure to bright light have already been examined and generally accepted [35]. A few studies have also provided evidence of the efficacy of cognitive-behavioral techniques in the treatment of chronic primary and comorbid insomnia [35]. One of the most popular ways of reducing the impact of shift work on occupational wellbeing is flexibility in organizing time schedules. In fact, flexibility/time reduction can be an advantage in sleep control and related health problems, but only if the latter is not linked to productive problems, otherwise the stress associated with the loss of work becomes a major risk factor [36]. A review of the literature indicates that interventions that increase control over work time and the decision latitude of workers through the introduction of flexibility have a positive effect on health. On the contrary, interventions related to organizational needs such as downsizing and involuntary reductions in working time have negative or equivocal effects [36]. In fact, other studies, such as the one recently conducted in the United States showing that persons working 52 h per week or more for a minimum of 10 years had a higher risk of poor self-reported general health, cardiovascular disease, and cancer [37] demonstrate that the real risk factor is not the work itself, but its inappropriate organization or its oppressive character. Once more, it is not the change in a parameter that can improve life, but a whole set of psychosocial factors associated with that parameter.

### 2.4. Loneliness

Numerous other individual factors affect sleep; one of these is loneliness. This is a well-known parameter for work psychologists who have developed numerous tools for measuring the feeling of solitude and social isolation (for example, UCLA Loneliness Scale [38], Existential Loneliness Questionnaire [39], Short Scale for Measuring Loneliness [40]). In the field of occupational medicine, social support (the opposite of isolation) is measured by the demand-control-support (DCS) model of Karasek [41]. The concept of peer- and superior- support is also included in the British HMS standard, measured by the HMS-RIT questionnaire [42]. Workers who have little social support suffer more than others from the effects of work stress (the so-called iso-strain condition, considered to be the highest risk of stress-related disease in the DCS model) and have fewer resources to help them recover when they are exposed to stressful life events. We must remember that over the years the effectiveness of certain social support networks for workers, such as trade unions, political parties and labor associations has been diminishing. It is not yet possible to say whether the “social media” that have largely replaced these organizations are as effective in reducing isolation in workplaces.

Social and emotional solitude were significantly associated with the difficulty of starting and maintaining sleep (DIMS) in a cohort of over 12,000 Norwegian students [43]. This study demonstrated that while the relationship between DIMS and social isolation was entirely attributable to the associated psychosocial stress, emotional solitude had a direct relationship with DIMS that did not depend on stress.

The feeling of loneliness was associated with poor quality sleep not only in the person affected by this condition, but also in his/her partner [44]. Confirmation of the association between loneliness and poor sleep quality was found in the Longitudinal Twin Study, a cohort study of 2232 twins born in England [45]. In this case, sleep disorders were evident, especially in young people who had suffered trauma or violence, and underlined the importance these traumas may have on the function of dreaming.

Loneliness has been associated with sleep disorders in numerous studies. A meta-analysis of six studies showed that the most evident insomnia symptoms were associated with nightmares and anxiety, but not with depression Insomnia is associated with loneliness, and this association can be explained by depression [46]. In the elderly, loneliness is generally associated with the use of antidepressants and benzodiazepines [47]. In the Danish case study “2013 Public Health Survey: How are you?”, loneliness in the elderly is associated with cardiovascular disorders, diabetes and migraine. Poor quality sleep facilitates these associations [48].

Social isolation at the workplace can be experienced by a number of professional categories. For example, it is often reported by top managers [49], although empirical studies do not confirm this evidence [50]. It is also common in minorities, such as migrant women [51].

A review of the literature confirms the existence of close ties between perceived social isolation and cardiovascular, neuroendocrine and cognitive function damage. Social isolation leads to depression, cognitive decline and sleep problems. There is a complex interrelation between social isolation and these neuro-psychological disorders [52]. Sleep disorders play a central role in this etiopathogenetic relationship. Studies conducted in an industrial work environment showed that psychosocial work factors, and particularly lack of social support and work-life conflict, play a key role and need to be taken into consideration in research and workplace health promotion [53].

### 2.5. Workplace Violence

Workplace harassment is significantly associated with sleep loss in US workers [54]. Also French studies showed that workplace bullying might be strongly associated with sleep disturbances [55]. Workplace injustice, violence and unwanted sexual attention are associated with increased frequency of sleep disorders among Korean employees [56]. Self-perceived justice, indeed, may be a protective factor against the effects of workplace violence on sleep [57]

Bullying or mobbing is associated with early awakenings and lack of resting during sleep, as shown by longitudinal WBH and PRISME studies [58]. In fact, bullying and unwanted sexual attention is prospectively associated with long-term sickness absence, and this association is partially mediated by poor sleep [59].

### 2.6. Acute and Chronic Psychosocial Stress

The central nervous system response to acutely stressful life events (not to be confused with the common stress of daily life) may vary from an effective response (resilience) to post-traumatic stress disorder or the onset of chronic mental illness. This response involves a series of morphological and neurochemical modifications including oxidative stress, which is an imbalance between the production of oxidizing species and antioxidant response [60].

Post-Traumatic Stress Disorder (PTSD) consists of a complex set of somatic, affective, and behavioral effects that are the result of a psychosocial trauma. It is characterized by nightmares, intrusive thoughts and flashbacks of the traumatic events, hypervigilance and sleep disturbances, and leads to a significant impairment of social life and interpersonal relationships. In some professional categories, such as military, policemen and night watchmen, emotional trauma is an occupational hazard.

Not all subjects who experience trauma undergo PTSD. In Canada, 76% of the population reported exposure to highly stressful events in 2013, but the prevalence of PTSD over the lifetime was estimated at 9.2%. In the Canadian military, this prevalence was greater than in the general population, 11.1% over the lifetime, and 5.3% in the previous year’s survey [61].

People who suffer from PTSD have sleep disturbances. Although the exact mechanism linking PTSD and sleep disorders is not fully understood, it has been suggested that there is a complex interplay between sleep fragmentation and neuroendocrine pathways. The existence of symptoms overlapping between PTSD and sleep disordered breathing poses diagnostic challenges [62].

In Vietnam veterans, PTSD severity and nightmare-related distress were correlated with increased frequency of awakening, shorter sleep duration, increased sleep latency and greater nightmare frequency. The perception of stress factors in daily activity was also associated with an increase in the number of nightmares, distress associated with nightmares and difficulty in falling asleep [63].

A significant co-morbidity of PTSD is drug abuse; sleep disturbances can facilitate these two pathologies. In fact, insomnia, nightmares and daytime sleepiness that are typical of PTSD can lead to drug use. However, the prolonged use of drugs may induce even more severe sleep disturbances and the development of tolerance and abstinence syndrome [64]. Work-related stress and sleep are closely related. Experience in workplaces indicates that not only major events but also modest occupational trauma, such as accidents or verbal aggression, may be associated with sleep disturbances [65]. Exposure to chronic stressors, including organizational, physical and psychological danger and lack of support, may adversely affect sleep quality in police officers [66]. In doctors on night duty the quality of sleep and psychosocial problems are significant predictors of the appearance of gastrointestinal disorders [67]. Hospice nurses at high risk for compassion fatigue report moderate-to-severe sleep disturbances that may be improved with cognitive-behavioral therapy [68]. A significant relationship between work-related stress and sleep disturbances was also observed in the Italian cross-sectional SSS survey (stress, sleep and metabolic syndrome) conducted on over 2000 healthcare and social workers. Preliminary data indicate that both stress and sleep disorders are associated with metabolic syndrome [69].

### 2.7. Gender

Gender differences have been identified in the response to environmental factors that may interact with sleep in the workplace [70]. A systematic review of epidemiological evidence revealed female gender, depressed mood, and physical illness as general risk factors for the onset of sleep disorders in later life, although specific physiological pathways have not yet been established [71]. There is, however, a growing recognition of sex disparities in sleep and rhythm disorders. Clinical research implicates a role for sex steroids in sleep modulation [72]. Gender differences in levels of neurotrophic factors regulating sleep have also been demonstrated [73]. Sleep disturbance is common during the menopausal transition. Gender severely interacts with age in sleep disorders of older workers [74] However, research on gender differences in sleep in occupational medicine is still limited, and more research is needed to determine the precise mechanisms through which gender-related factors influence sleep over time in occupational cohorts [75].

### 2.8. Cultural and Behavioural Factors

Modern society requires round-the-clock activity. The social context has an impact not only on what we think about sleep, but also on how we control or self-regulate our habits [76]. Interviews with shift workers and students, two active day-to-day categories, indicated that all of them considered sleep to be of prime importance for health, overall wellbeing, physical appearance, and the ability to work on a cognitive and physical level. Nonetheless, the time dedicated to sleep is often consciously reduced due to work demands and social activities.

The two categories also had different attitudes toward sleep. Shift workers generally strived to achieve adequate sleep time within the 24 h [76]. Students, on the other hand, emphasized the importance of adopting flexible sleeping hours in order to maintain social relationships despite study commitments. Respondents reported using a large number of strategies, techniques, practices and technologies to overcome or delay daytime sleepiness and stimulate or promote vigilance at socially desirable times. Although these strategies sometimes seemed effective for immediate needs, nevertheless, the resulting reduction in sleep remained a problem for those who were exposed to it. All the respondents continued to carry out their activities with chronic quantitative or qualitative sleep debt [76].

## 3. Part II. The Effects of Sleep on Health

### 3.1. Sleep Disorders and Quality of Life

Numerous studies report the existence of a significant association between obstructive sleep apnea (OSA), insomnia, restless leg syndrome (RLS) (the three most common sleep disorders) and the quality of life.

Sleep apnea is the most common cause of excessive sleepiness. This disorder leads to significant impairment in the quality of life and cognitive performance, especially if it is associated with obesity [77,78]. A meta-analysis carried out in 2008 demonstrated that CPAP in OSA patients did not improve general QOL scores but did improve physical domains and vitality [79], while a more recent meta-analytic study has found a small average improvement in both mental and physical component scores in treated patients [80].

In cross-sectional and short-time longitudinal studies chronic insomnia has been associated with a poor quality of health [81], not only in obvious domains such as vitality and energy/motivation, but also in other mental, social, and physical activities such as work performance, cognitive functioning, emotional regulation, and relationship/family functioning [82]. Insomniacs report difficulties with cognitive, emotional, and physical functioning, reduced work performance and social participation, a feeling of isolation and limited life aspirations [82]. Treatment for insomnia may improve the quality of life [83]. Several extensive surveys observed that the worst is insomnia, worse is the quality of life [84,85,86], and with age [87]. Improving sleep by means of both pharmacological and non-pharmacological intervention can lead to significant improvements in HRQoL domains [82,87]. Restless leg syndrome (RLS) also impairs mental and physical functioning [88,89]. Treatment with exercise and drugs proved to be effective in improving the quality of life [90].

### 3.2. Sleep and Mental Health

Good sleep guarantees wellbeing and mental health. A longitudinal study conducted on 969 Japanese workers found that the participants who felt rested after sleep had a significant chance of manifesting mental wellbeing after three years of follow-up [91].

In studies on work populations that by definition are composed of healthy subjects, neuropsychological pathologies are frequently diagnosed in workers who have problems with the quantity or quality of sleep. For example, in a study of 1134 Brazilian nurses, a significant association was observed between night work (5 or more shifts in 2 weeks) and the frequency of common mental disorders (anxiety, depression) [92].

A recent meta-analysis confirmed evidence in the literature indicating that OSA patients have a high prevalence of common chronic psychiatric disorders such as anxiety and depression. In patients receiving treatment, the prevalence of depression was 31.7% (95% CI: 24.0–40.0) and anxiety 35.7% (95% CI: 24.5–47.8) [93]. A less well-known fact is that the level of wellbeing also deteriorates in individuals who suffer from pre-clinical, self-diagnosed sleep disorders. A 2014 study conducted in Italy demonstrated that workers with OSA report a level of psychological wellbeing below that of their healthy colleagues [94]. In an Australian cohort of more than 13,000 working-age males, self-diagnosed OSA had a prevalence of between 2.2% and 7.8% in the various age groups and was significantly associated with poor subjective wellbeing and reduced work ability, concentration and memory, as well as with cardiovascular risk factors [95]. The association between poor quality/quantity of sleep and psychological illness was confirmed in a 2017 study on professional drivers commissioned by the Italian Ministry of Transport [96].

Effective OSA treatment should lead to improvement in common mental disorders (CMDs) such as anxiety and depression. However, a review of the evidence has shown that treatment has a moderate clinical effect on symptoms of depression and anxiety in OSA, but is not more effective than a placebo. This suggests that the improvement in symptoms may be due to patient expectations and contact with healthcare providers [97].

Poor sleep and chronic insomnia are associated with impulsiveness in patients with borderline or antisocial personalities [98]. This means that poor sleep hygiene in workplaces can result in destructive behavior in these categories of workers.

### 3.3. Sleep and Cognitive Function

Maintaining a good cognitive level in workers is a public health priority, given the aging of the workforce [99]. Numerous studies have attempted to clarify the relationship between disordered sleep and neurological function. Recent studies on superior animals have shown that the hypothalamus is a bidirectional integrator of peripheral humeral and neuronal information that influences brain aging which, in turn, sets the pace for general aging [100]. A decline in the nervous system produces gradual cognitive and motor impairment, and affects social correlations. Only in recent years has the role of neuro-microglial networks and sirtuines (a class of NAD + dependent deacylase) been clarified in an attempt to prevent brain deterioration. Sleep, in fact, plays a crucial role in brain aging [101]. Many neurodegenerative diseases such as Alzheimer’s disease, Parkinson’s, and Huntington’s chorea have the following symptoms in common: an alteration in circadian rhythms, impaired behavior, destruction of the sleep/wake rhythm and physiological processes, a reduction in neuro-mucous growth and bio-metabolism with an increased oxidation production [102]. Circadian rhythm disorders are among the early symptoms of these diseases and the molecular mechanisms of the circadian system probably play a decisive and perhaps causal role in the evolution of neurological diseases [102,103].

OSA-the commonest sleep disorder in workers-has frequently been linked with cognitive decline. Sleep disorders in older workers, in people with mild cognitive impairment and with neurodegenerative diseases may be the first sign of the onset of dementia. Cognitive impairment may be a sign of an un-diagnosed sleep disorder [104]. OSA has many risk factors in common with Alzheimer’s disease and may be part of the latter’s pathological process [104]. The occurrence of sleep disorders in older workers should induce the work physician to promptly inform his/her primary care colleagues. The belief that older persons sleep less than young people is not based on a real physiological need of less rest, but is indicative of a pathological, pre-clinical condition. Sleep disturbances are more common in the elderly because multi-morbidity, polypharmacy, primary sleep disorders and psychosocial factors that alter sleep are highly prevalent in this age group [105].

A quantitative analysis of the association between OSA and neuropsychological performance showed a small negative relationship, and it is likely that some older adults may be at risk of cognitive impairments attributable to OSA; however, the connections between OSA and cognitive ability in working life vary considerably and the risk of bias renders evidence inconclusive [106]. According to the official consensus statement of the American Thoracic Society Research, there is no conclusive evidence that mild OSA leads to significant neurocognitive and cardiovascular complications [107].

### 3.4. Sleep and Response to Stress

Since the harmful health effects of lack of sleep are well documented, scientific research has focused on possible pathogenic mechanisms. One of these hypothetical mechanisms involves the correlation between sleep and physiological response mechanisms to external stimuli.

The increase in cortisol due to acute psychosocial stress varies in relation to the quality of sleep, especially in men. In addition, daytime sleepiness is associated with a reduced response to stress [108]. Laboratory studies indicate that sleep deprivation increases allostatic responses to stress [109].

In premenopausal women, hormonal changes, flushes and psychosocial factors contribute to increasing the frequency of insomnia. It has been demonstrated that there is more electro-encephalographic activation and a lack of vascular activity recovery after acute stress in women with insomnia, indicating that the latter contributes to increasing stress sensitivity [110].

### 3.5. Sleep, Stress and Cardiovascular Risk

Stress and sleep disorders are important cardiac risk factors. Regular sleep rhythms and schedules are more important than sleep duration for health and wellbeing, because the latter may vary from one individual to another.

A two-year longitudinal study of 99 subjects (mainly nurses) found that work-related stress and sleep deprivation are associated with an increase in cardiovascular risk factors in individuals who do not exercise [111].

Sleep disorders are also associated with components contributing to metabolic syndrome, e.g., obesity. Obesity is a multifactorial condition influenced by genetic, environmental, and lifestyle factors. A study of 119,859 white European adults indicated that some sleep characteristics (sleep duration, chronotype, sleeping habit, shift work) change the phenotypic expression of obesity. This study showed that the association between genetic risk of obesity and phenotypic measures (BMI, abdominal circumference) is intensified by poor sleep quality [112].

In our own investigation [113] of 918 workers in a large Roman hospital, occupational stress, measured as effort/reward imbalance (ERI) [114], was significantly associated with metabolic syndrome (OR = 1.67; 95% CI: 1.17–2.38). After correction for demographic variables, smoking, social support, over-commitment, work disturbance and mental health (anxiety/depression), the significance of the association remained unchanged (OR = 1.57; 95% CI: 1.04–2.38). Occupational stress was also associated with hypertriglyceridemia OR = 1.52; 95% CI: 1.07–2.16) and obesity (OR = 1.38; 95% CI: 1.07–1.79). Correction for confounding factors failed to alter the strength of these associations. On the contrary, sleep disturbances strengthened the association.

The study we conducted on a police flying squad [115] that followed a controlled diet and did regular and rigorous physical activity showed that work-related stress leads to increased metabolic and cardiovascular risk. After 5 years of observation, policemen who had always been classified in the highest quartile of perceived stress had higher levels of triglycerides and lower levels of HDL cholesterol than colleagues who performed the same tasks but were in the lower quartile of stress. Patients with the highest perceived stress had an increased risk of contracting metabolic syndrome (aOR = 2.68, 95% CI: 1.08–6.70) and hypertriglyceridemia (aOR = 7.86; 95% CI: 1.29–48.04). Among the stress-measuring variables, the most significant predictors of metabolic syndrome were psychosocial occupational load (Demand) and the effort needed to perform the job (Effort).

The association between stress and metabolic syndrome increased when sleep was taken into account. In the same cohort, at baseline, the police officers in the highest stress quartile had a higher frequency of sleep disturbances than the policemen with a lower stress level, but there were no substantial differences in metabolic values. At follow-up, sleep disorders in the highest quartile had significantly increased. In addition, all metabolic parameters were significantly worse in those subjects who had experienced the highest levels of stress in the five-year period than in the most resilient group. In conclusion, policemen suffering from stress and sleep disorders had a significantly greater risk of metabolic syndrome than their less stressed colleagues with good sleep habits.

The association between stress and age and sleep disturbances was studied in a sample of over 900 offshore platform operators. Age and stress increase or multiply the effects. The duration of sleep is mathematically predicted by the quadratic value of the age multiplied by job demand; the quality of sleep is proportional to age and job demand [116].

The relationship between age, stress and sleep disturbances varies during different stages of life. A Scandinavian longitudinal study of backbone and psychosocial factors demonstrated that stress, life dissatisfaction and sleep problems are significant predictors of back pain in forty-year-olds, while in the young backache is associated only with the physical characteristics of work (load to be moved, incorrect posture, vibrations) [117].

The relationship between work stress and sleep disturbances in older workers is particularly complex due to overlapping stress factors and dissatisfaction with life. In a Japanese case, depression and a feeling of unhappiness were significantly associated with age and reduced sleep duration, even after adjusting for confounding factors [118].

Destruction of the sleep cycle is associated with increased activity in the sympathetic nervous system and the hypothalamic-pituitary-adrenal axis, with metabolic effects, circadian rhythm changes and pro-inflammatory responses. In healthy adults, short-term sleep interruptions result in a greater response to stress, an increase in painful musculoskeletal sensitivity, and a decrease in the perceived quality of life. There may also be a state of emotional distress, and mood disorders, memory deficits and cognitive and performance disorders may appear. Long-term consequences in otherwise healthy individuals include hypertension, dyslipidemia, cardiovascular disease, weight loss, metabolic syndrome, type 2 diabetes, and colon cancer. Interrupting the sleep cycle also increases all-cause mortality. In subjects affected by other morbid conditions, destruction of the circadian rhythm may reduce the quality of life [119].

It is useful to remember that not only lack of sleep, but also sleeping too long constitutes a health risk factor. The prospective study of the European Prospective Investigation into Cancer-Norfolk cohort that followed 9692 adults aged 42–81 for 9.5 years demonstrated that long-term sleep is associated with increased risk of stroke (OR = 1.46; 95% CI: 1.08–1.98). On the contrary, short-term sleep was not significantly associated with risk in this cohort [120]. A meta-analysis conducted by the same authors confirmed that there is an increase in vascular risk for long-term sleep (OR = 1.45; 95% CI: 1.30, 1.62) and there may be a smaller increase of the vascular risk for short-term sleep (OR = 1.15; 95% CI: 1.07, 1.24) [120].

Long-term sleep is also associated with mortality for all causes according to National Health and Nutrition Examination Surveys carried out from 2005 to 2008. Daytime drowsiness and inadequate sleep quality are associated with increased mortality for all causes, especially in the elderly [121].

### 3.6. Sleep and Accidents

The presence of undiagnosed sleep disturbances can lead to significant and misunderstood health and safety risks. One out five vehicle accidents is related to sleepiness [122]. The CNH-Iveco Study conducted on Italian roads [123] showed that about a quarter of truck drivers (24.3%) have a sleeping debt of over two hours; more than a quarter (25.4%) have a high risk of obstructive night-time apnea (OSA); 13.4% suffer from excessive daytime sleepiness (EDS); and 27.5% suffer from insomnia. More than one in three (34.8%) reported a driving accident in the previous three years, and 10% had had a near-miss accident in the six months before examination. An analysis of these data has shown that OSA, sleep debt and EDS are independently associated with road accidents and near-miss accidents. The road safety of drivers, but also of third parties, depends on sleep hygiene and the elimination of factors that contribute to the current situation, such as excessively long sedentary periods, inadequate eating habits, stress, and inadequate working hours.

The significant consequences of a high frequency sleep disorder such as OSA (which often goes undiagnosed or untreated) do not affect road safety alone. A recent meta-analysis revealed that the risk of accidents at work more than doubles in the presence of OSA (OR = 2.18; 95% CI: 1.53–3.10). Drivers with OSA have an even heavier increased risk of occupational non-driving injury (up to 15 fold) [124]. Since this morbid condition is so prevalent, we can estimate that in Italy early identification and treatment of OSA in workplaces could prevent up to 1 million work accidents, 1000 deaths and 20,000 cases of permanent disability every year [125]. According to another meta-analysis of 27 observational studies, approximately 13% of work injuries could be attributed to sleep problems [126].

Insomnia is also an independent risk factor for road accidents and near-miss accidents [127]. Short naps protect against road accidents more than breaks. Short sleep duration, in itself, is not a risk factor. This is because there are some individuals who need less sleep and are likely to find an advantage in carrying out this type of occupation.

## 4. Conclusions

Over the last 50 years, technological advances and prevention have greatly reduced the level of chemical and physical hazards in the workplace. However, productive and social requirements have increased the demand for shift work and working up to at an advanced age of life. These and other psychosocial and individual work-related factors prevent workers from maintaining a good level of sleep.

Duration of sleep and the circadian cycle are related to occupational fatigue and daytime sleepiness, which can lead to a variety of adverse medical outcomes. In 2012, the American College of Occupational and Environmental Medicine issued a set of guidelines for fatigue prevention [128]. In Italy, a proposal was put forward for a Sleepiness Prevention System (SPS) [129]. Measures suggested for mitigating fatigue and the risk of sleepiness in the workplace include adequate staffing, correct shift scheduling, work environment design, employee fatigue training, sleep disorder screening and management, and sleepiness and fatigue monitoring. Experience has shown that the screening of sleeping disorders and intervention to determine poor sleep quality can be incorporated into current health surveillance activities without a significant increase in medical costs or lost of medical time, and result in a substantial improvement in the quality of health care [94,130]. A number of health promotion programs targeted at sleepiness and fatigue prevention have been implemented [131,132,133,134,135] and proved to be effective; other programs, however, have had negative results [136,137]. In many studies both the internal and external validity of findings have been limited and the range of factors investigated has been insufficient. Only a few studies have investigated more complex interactions between different factors [138].

Offering a healthy sleep program through a worksite wellness facility may avoid the medicalization of sleep, overcoming the common barriers to health care service. Healthy sleep hygiene, stress management and cognitive strategies for sleep, meditation techniques and movement exploration, increasing awareness of common sleep disorders and behavioral interventions to promote sleep may be included in a comprehensive health promotion program. Detailed action steps directed at key stakeholders can also encourage healthy behaviors such as regular exercise, smoking cessation, proper nutrition, socialization and work-life balance.

Sleep is an essential factor for the wellbeing of workers. Both employees and employers have a vested interest in maintaining and also improving a high standard of worker welfare. Employers have immediate benefits in terms of higher productivity, better product quality, and reduced conflict and absenteeism/presenteeism. Increasing worker wellbeing, work engagement and job satisfaction is the most effective way of counteracting absenteeism and presenteeism and, in accordance with current needs, it also enables workers to be active and productive even at an advanced age [139].

Occupational health must change its traditional role of preventing occupational diseases caused by chemical and physical agents so that instead of treating illnesses it can develop health promotion activities designed to promulgate healthy life styles and increase wellbeing. Knowledge derived from top-quality workplace studies will provide support for future intervention programs.
